# Editorial: Horizontal transfer of antibiotic resistance genes in the environment: dynamic, contributing factors, and control

**DOI:** 10.3389/fmicb.2025.1692478

**Published:** 2025-10-27

**Authors:** Umer Zeeshan Ijaz, Yong Qiu, Xiaoqin Zhou, Huabing Yin, Bing Li

**Affiliations:** ^1^James Watt School of Engineering, University of Glasgow, Glasgow, United Kingdom; ^2^School of Environment, Tsinghua University, Beijing, China; ^3^School of Energy and Environmental Engineering, University of Science and Technology Beijing, Beijing, China

**Keywords:** antibiotic resistance genes (ARGs), horizontal gene transfer (HGT), environmental pathways, transmission, pathogen

One of the most urgent public health emergencies of our day is the spread of antimicrobial resistance (AMR) throughout the world. It compromises the effectiveness of antibiotics, making it more difficult to treat common infections and threatening human's health. The articles compiled here provide a comprehensive and multifaceted perspective on antibiotic resistance, covering the factors and mechanisms driving horizontal transfer of antibiotic resistance genes (ARGs), ARG dissemination and its control, and the emergence of resistance in certain pathogens. Collectively, they highlight the complex biological processes, environmental pathways, and significant clinical advancements that emphasize how urgent it is to confront AMR. The mechanisms of resistance gene transfer, the environmental and agricultural spread of these genes, and the characterization of resistance in particular contexts are the three main issues under which this editorial arranges these pieces.

## Mechanisms of antibiotic resistance gene transmission

The complex process of ARG transmission via horizontal gene transfer (HGT) is a major and recurrent theme. An overview of the main HGT pathways: conjugation; transformation; transduction; and the more recently identified vesiduction, is given in a review by Liu et al.. The article's methodical overview of the approaches and mathematical models currently being used to measure and forecast these transfer events gives it rigor and serves as a solid roadmap for further study in this field.

By concentrating on vesiduction, Xu et al. offer a particularly original viewpoint. They show that outer membrane vesicles (OMVs) secreted from *Actinobacillus pleuropneumoniae* can successfully transmit the *floR* resistance gene to other bacteria, particularly Enterobacteriaceae. Because it reveals a hitherto overlooked route for ARG spread, this study is important because it raises the possibility that vesiduction contributes more to interspecies resistance transfer than previously believed.

By examining how mobile genetic elements (MGEs) in *Klebsiella pneumoniae* serve as carriers of both virulence and resistance genes, Han et al. summarizes MGE types and how they contribute to the establishment of harmful strains such as carbapenem-resistant hypervirulent *K. pneumoniae* (CR-hvKP). The concept of MGEs building intricate, “Russian doll”-like structures offers a new perspective: it provides an elegant explanation for the co-acquisition of several features that increase a pathogen's pathogenicity and fitness.

## Environmental and agricultural dissemination of ARGs

The Research Topic also investigates the ways in which ARG dissemination in diverse settings. According to Zhao et al., hospital sludge water is a crucial conduit for the introduction of pathogenic bacteria and ARGs into the broader ecosystem, establishing a direct connection between clinical settings and the environment. This study is significant because it clearly documents the relationship between hospitals and the environment, highlighting the necessity of better waste management practices to control resistance.

Liu et al. offer an investigation of multi-drug resistant *Proteus mirabilis* obtained from pigs in an agricultural setting, concentrating on the IncQ1a plasmids that cause this resistance. A thorough genetic and phenotypic research that yields an accurate map of the resistance genes present in animals. In order to address this Research Topic, Shi et al. provide a unique and useful solution: using compost that has been altered with biochar to reduce the spread of ARG and enhance soil fertility in agricultural soil, providing a two-pronged approach to sustainable agriculture.

By demonstrating that natural substances, particularly short-chain fatty acids (SCFAs), can prevent bacterial plasmid transfer *in vitro* and in *ex vivo* chicken tissue, Ott and Mellata provide an innovative remedy. The finding is quite innovative since it provides a non-antibiotic method of controlling resistance in animals that produce food. However, further research is warranted to determine whether SCFAs can inhibit HGT across diverse environmental contexts.

## Characterization of pathogen-specific resistance

The formation of mosaic *penA-60* and *penA-237* alleles in a core genogroup of *Neisseria gonorrhoeae* is presented by Thomas et al.. The loss of susceptibility to extended-spectrum cephalosporins, a family of antibiotics that were among the final available treatments for gonorrhea, is documented. This study serves as a clear reminder of the importance of HGT in AMR spread, as independent recombination events in *Neisseria subflava* and *Neisseria sicca* may have led to the generation of mosaic *penA-60* and *penA-237*.

The spread of antibiotic resistance genes among *Campylobacter jejuni* is a serious problem. Qiu et al. then provides a fresh examination into how the Type III restriction-modification (R-M) system in *C. jejuni* can limit the horizontal transmission of ARGs, which provides a theoretical basis for further investigating AMR acquisition dynamics in other bacterial populations, which may in turn inform the development of new strategies for addressing the drug resistance problem.

## Conclusions

A vital “take-home message” for the field of AMR is highlighted by these articles, which span a variety of research fields from molecular genetics to environmental science and clinical microbiology: AMR is a complex, interconnected, and dynamic problem that necessitates a multifaceted, holistic approach to combat. From thorough molecular characterizations to innovative therapies like biochar and short-chain fatty acids, these studies offer a path forward for further initiatives. It is obvious that effective remedies need to take into account the complete “resistome” including the environmental elements such as emerging contaminants (i.e. microplastics) and bacterial defense mechanisms that affect its spread. The most promising approach to combating AMR is to adopt an integrated viewpoint that links the macroscopic issues of sustainable agriculture and public health with the microscopic world of bacteria and ARGs.

